# Physiological mechanisms and adaptation in high-performance shooting: a biomarker review

**DOI:** 10.3389/fnhum.2026.1901170

**Published:** 2026-09-17

**Authors:** Larisa Bayova, Nikita Grigorev, Victor Kazantsev, Susanna Gordleeva

**Affiliations:** 1 Center for Cognition and Decision Making, Institute for Cognitive Neuroscience, HSE University, Moscow, Russia; 2 Laboratory of Neurodynamics and Cognitive Technologies, Neuroscience Research Institute, Lobachevsky State University, Nizhny Novgorod, Russia

**Keywords:** biomarkers, electroencephalography, heart rate variability, high performance, neurofeedback, quiet eye, sport shooting

## Abstract

Precision shooting depends on the coordinated integration of multiple psychophysiological systems. This review summarizes the current literature on quantifiable biomarkers associated with high performance in shooting sports, examining neural, cardiac, oculomotor, muscular, postural, and respiratory activity. Special attention is given to individual variability in postural control and sensorimotor integration as key factors distinguishing expert from novice shooters. Electroencephalographic studies show that experienced shooters exhibit characteristic patterns of cortical activity, including left-hemisphere alpha synchronization reflecting reduced verbal-analytic processing, specific theta rhythm dynamics associated with focused concentration, and increased sensorimotor rhythm power associated with efficient movement preparation. Cardiac activity studies demonstrate that successful shooting is associated with a slower heart rate, a specific shot execution time within the cardiac cycle, and increased heart rate variability reflecting parasympathetic dominance. Oculomotor activity determines the duration of a steady gaze, a critical factor in accuracy, especially under competitive pressure. Electromyographic analysis shows that experts maintain stable, low-level muscle activation compared to novices, who exhibit a progressive increase in tension. Postural balance plays a significant role in weapon stability. Breathing regulation facilitates synchronization with exhalation and an optimal state of the autonomic nervous system. Despite the significant contribution of each parameter to performance, most studies examine individual parameters in isolation. Future research should focus on a multimodal approach that considers intersystem interactions and integrates relevant analytical tools, such as neural networks and explainable artificial intelligence. Training approaches using biofeedback have been shown to accelerate skill acquisition. Therefore, the development of personalized real-time feedback systems that transform raw physiological data into practical recommendations will be essential for optimizing training effectiveness and achieving maximum shooting performance.

## 1 Introduction

Sport shooting is a complex sensorimotor task that requires visuospatial attention and precise motor control (Harris et al., 2023). Performance in this discipline depends not only on technical factors, such as stance stability and aiming accuracy, but also on the athlete's psychophysiological state (Wang et al., 2023). This has led to growing interest in identifying quantifiable neural and physiological markers that distinguish successful from unsuccessful performance. The relevance of such biomarkers is highlighted by the observation that even among highly trained professionals, performance varies considerably, indicating that extensive experience alone does not guarantee success (Marineau et al., 2024). In the present study, we operationalize the term “biomarker” as a quantifiable physiological metric that is systematically associated with, and can serve as a predictor of, positive changes in performance.

Advances in monitoring technology now allow simultaneous recording of multiple physiological signals during ecologically valid shooting tasks (Badarin et al., 2023). Electroencephalography (EEG) records cortical electrical potentials generated by synchronous postsynaptic activity of large neuronal populations (Binnie and Prior, 1994). Electrocardiography (ECG) provides measures of heart rate, RR intervals, and heart rate variability (HRV) (Lanfranchi and Somers, 2011; Ware et al., 2021). Eye movements and gaze behavior are captured using electrooculography (EOG) or video-based eye tracking, yielding metrics such as blink rate and quiet eye (QE) duration (Creel, 2019; Punde et al., 2017). Electromyography (EMG) records the bioelectrical activity associated with muscle contraction (De Luca, 2006). Postural sway and weapon stability are measured with force platforms and optical tracking systems (Mononen et al., 2007). Respiratory parameters, including rate and vital capacity, are obtained using thoracic sensors and spirometry (Ertürk et al., 2023). Together, these methods as shown on Figure 1 provide complementary perspectives on the shooter's physiological state.

**Figure 1:**
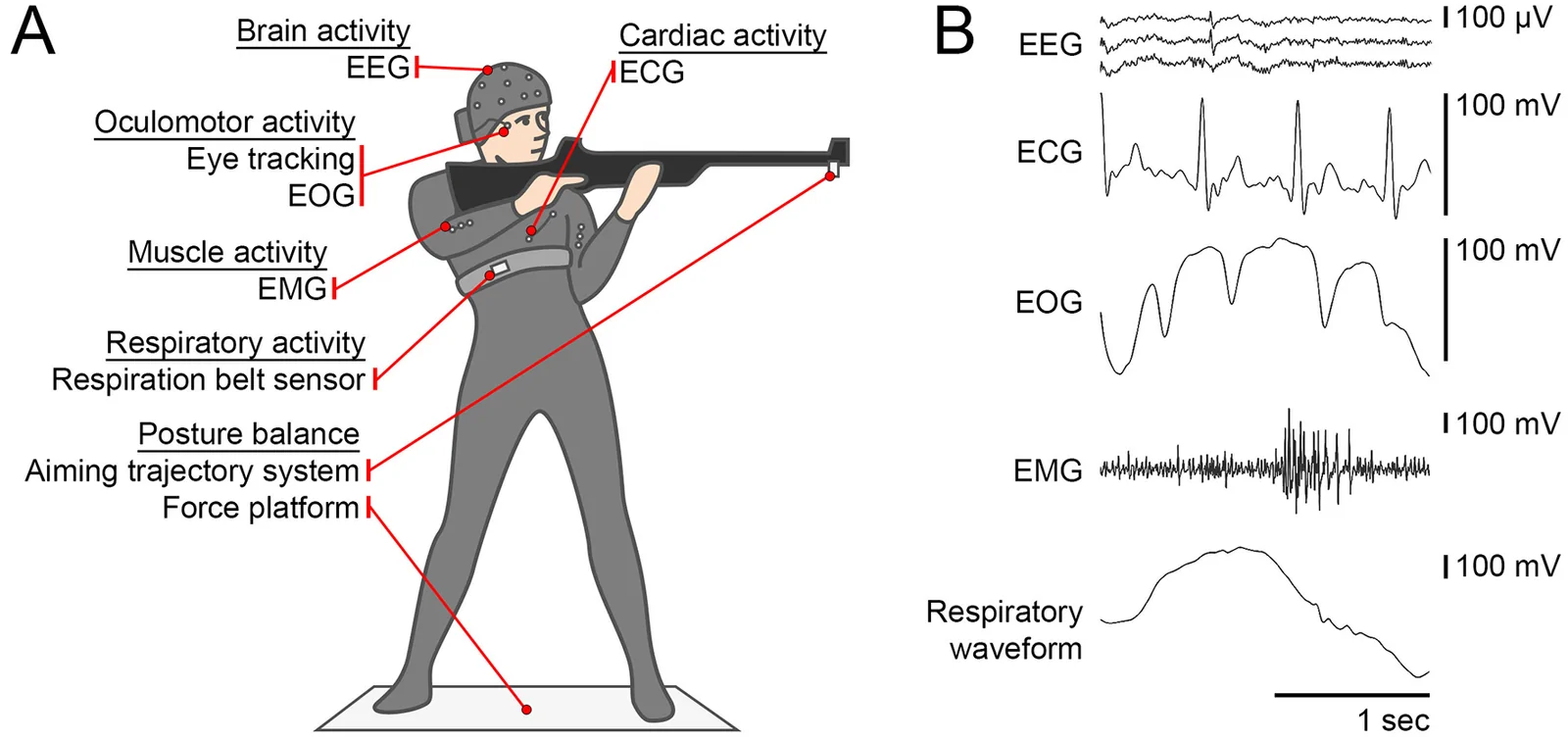
Modalities studied in shooters and methods of their registration in environmentally correct conditions. **(A)** Methods of simultaneously registering activities during shooting. **(B)** Graph demonstrating parallel recording of multimodal raw signals. ECG, Electrocardiography; EEG, Electroencephalography; EMG, Electromyography; EOG, Electrooculography.

Using these techniques, early research often compared novice and expert athletes to identify expertise-related biomarkers (Janelle et al., 2000; Doppelmayr et al., 2008). Such cross-sectional studies have shown, for instance, that experts display more efficient cortical processing, consistent with the neural efficiency hypothesis (NEH), which proposes reduced brain energy expenditure with task mastery (Li and Smith, 2021; Hatfield et al., 2004). However, cross-sectional comparisons provide only a static view of the expert state and do not capture the dynamics of skill acquisition or trial-to-trial performance fluctuations. Longitudinal training studies and within-session comparisons of successful and unsuccessful shots address this limitation. These designs have revealed distinct cortical activity patterns preceding hits compared to misses, implicating the brain in real-time performance optimization (Cheng et al., 2023).

The growing research interest in the psychophysiology of sport shooting is reflected in the distribution of publications by keyword frequency, presented in Figure 2. Despite that, studies recording multiple categories of biomarkers simultaneously remain uncommon. Most research has examined single parameters in isolation, potentially overlooking interactions between physiological systems. This is an important gap, as shooting involves concurrent and sequential integration of neural, cardiac, respiratory, and motor processes. An example of a comprehensive multimodal approach was employed by Badarin et al. (2023), who concurrently recorded EEG, ECG, EOG, respiration, and fatigue measures in novice participants during shooting training. Performance improvements were accompanied by specific changes across physiological domains, including increased delta and right temporal alpha power before missed shots and correlations between respiration rate and shooting success. This study illustrates how multimodal designs can reveal inter-system interactions and provide a more complete characterization of the psychophysiological state underlying successful performance. Understanding individual variability in postural control and sensorimotor integration is particularly relevant for precision shooting, where small differences in these domains translate into meaningful performance differences.

**Figure 2:**
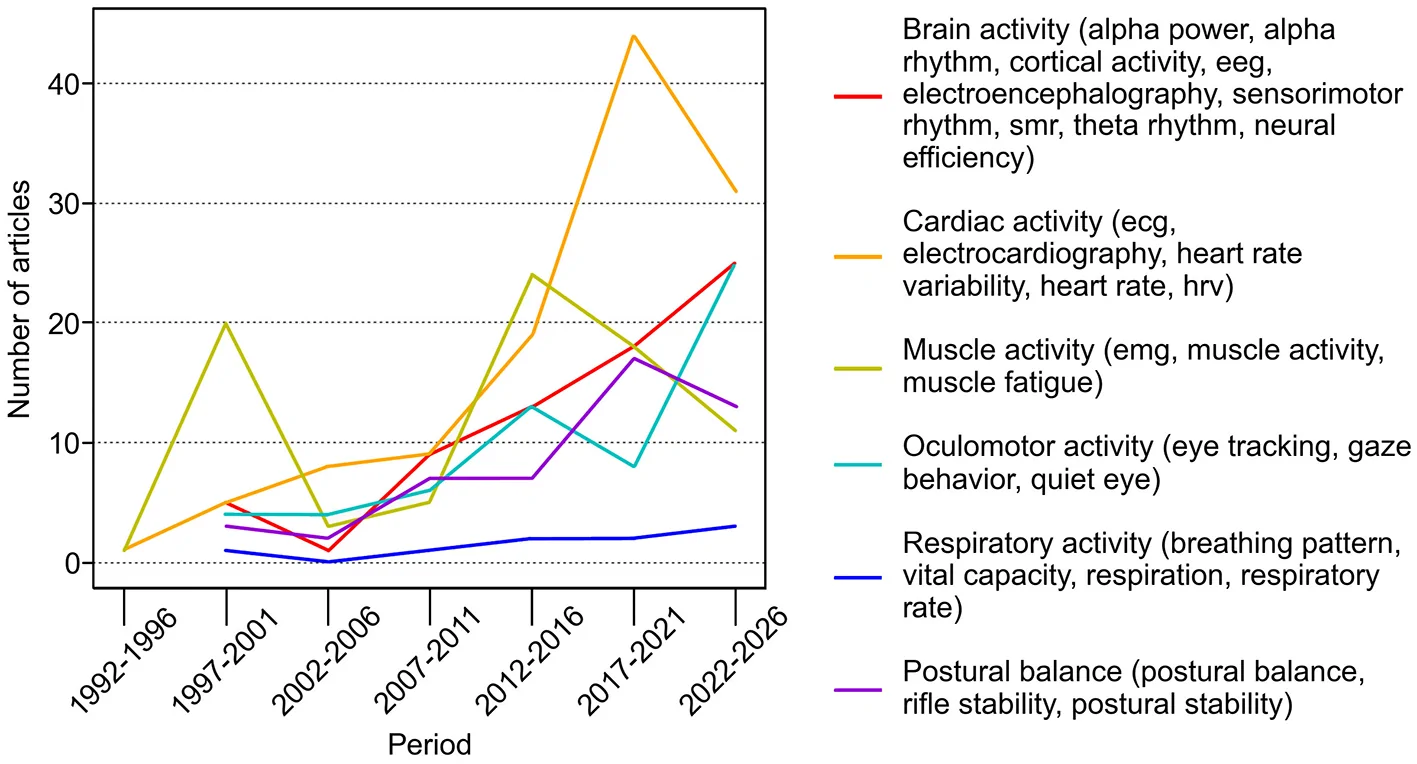
Distribution of publications by keyword frequency (based on a query “sport shooting”). ECG, electrocardiography; EEG, electroencephalography; EMG, electromyography; HRV, heart rate variability; SMR, sensorimotor rhythm.

Therefore, this review synthesizes current literature on psychophysiological biomarkers of high performance in precision sports. Subsequent sections examine biomarkers derived from various biological systems. Each section presents the recording methodology followed by empirical findings linking these measures to shooting performance. A final section addresses the effects of targeted training interventions, including those using biological feedback, on physiological parameters and performance outcomes. By integrating findings across these interconnected domains, this review aims to provide a comprehensive account of the psychophysiological foundations of elite shooting performance and to highlight the value of multimodal approaches for training optimization and performance monitoring.

For broader perspectives, readers are referred to several existing reviews (Corrado et al., 2024; Marineau et al., 2024; Harris et al., 2023; Li and Smith, 2021; Spancken et al., 2021; Nakata et al., 2010).

## 2 Methods

In accordance with the aim of this study, we searched Google Scholar and PubMed databases using the specific keywords presented in Figure 2. We reviewed studies published between 1992 and the present (2026). The inclusion criteria were: (1) relevance to the research topic and the biomarkers under consideration, and (2) publication in peer-reviewed journals. Additionally, we included a conference paper, as it presented unique methodological approaches relevant to our analysis.

## 3 Brain activity

The study of sport shooting provides a valuable perspective on how extensive motor practice shapes brain function. It is consistently demonstrated that prolonged training leads to characteristic changes in cortical activity. Professional shooters exhibit decreased activation in specific cerebral regions compared to novices, indicating that such changes emerge over time as a direct result of practice (Hatfield et al., 2004). This observation is consistent with findings from rodent models, which show that training induces long-term neuroplastic changes in the primary motor cortex, including synaptogenesis and topographic reorganization (Rioult-Pedotti et al., 2000; Kleim et al., 2002). It is therefore widely assumed that similar adaptive processes occur in sport shooters during skill acquisition, highlighting the importance of understanding the underlying physiological mechanisms (Nakata et al., 2010).

According to NEH, skill repetition leads to improved performance accompanied by a reduction in the brain's energy expenditure, while maintaining the same level of output (Li and Smith, 2021). This principle finds support in studies of cerebral glucose metabolism, where higher neural efficiency correlates with reduced glucose consumption (Haier et al., 1988). Functionally, this phenomenon is associated with the automation of motor tasks and a diminished need for conscious cognitive control. For instance, decreased activity in sensory and motor cortical areas is interpreted as evidence of more efficient neural processing with lower energy costs (Di Fronso et al., 2016). However, it is important to recognize that neural efficiency does not simply equate to a global reduction in cortical activation. Research has also documented selective increases in activity within key regions, such as the anterior cingulate cortex and medial prefrontal cortex, particularly during the critical preparatory moments before a shot. This suggests a more nuanced reorganization: rather than a uniform decrease, there is an efficient redistribution of neural resources, focusing energy on areas responsible for higher cognitive control when it is most needed (Doppelmayr et al., 2008). These patterns of brain activity are commonly investigated using EEG, which captures changes in neural oscillations and their derived measures, including event-related desynchronization (ERD) and synchronization (ERS).

Among the various EEG rhythms, alpha-band activity has received particular attention in shooting research due to its consistent association with expertise and performance quality. Studies show that the amplitude of alpha-band ERD is lower in professionals than in non-professionals, who tend to exhibit alpha ERD across the entire scalp. In contrast, professionals demonstrate high-frequency alpha-band ERS (10–12 Hz) immediately before a successful shot, with this ERS being more localized to right parietal and left central areas. This pattern suggests that experts engage in more selective activation of cortical populations during precise motor tasks (Del Percio et al., 2009). In this context, alpha ERS is interpreted as a marker of neural inhibition, while ERD reflects cortical activation (Klimesch, 2012); accordingly, a decrease in alpha rhythm (mu-ERD) indicates activation of task-relevant regions (Pfurtscheller, 2006). More specifically, successful performance in professional shooters is preceded by a marked increase in alpha power over the left temporal area in the 6 s before the shot, whereas no such change occurs over the right temporal area. Notably, this left-sided alpha synchronization—interpreted as suppression of verbal-analytical processing, a hallmark of automated skill execution—is absent during unsuccessful attempts (Wang et al., 2023). The phenomenon of left-hemisphere alpha enhancement during aiming in experts has been consistently observed, particularly in anterior temporal regions, and is thought to reflect reduced engagement of cognitive processes that could interfere with motor automaticity (Janelle et al., 2000; Loze et al., 2001). Furthermore, alpha power in the occipital area also fluctuates with performance quality: it increases before the best shots and decreases before the worst (Janelle et al., 2000; Loze et al., 2001). Professionals also exhibit greater hemispheric asymmetry in alpha power compared to non-professionals, and an increase in average alpha power over the right temporal area has been observed before missed shots (Loze et al., 2001). The competitive state itself modulates alpha activity, with changes in alpha power across anterior frontal, central, temporal, and occipital regions correlating with shooting accuracy (Zhang et al., 2021). Importantly, no such relationship between alpha ERS/ERD and performance is found in non-professional shooters, underscoring the specificity of these neural adaptations to expertise (Zhang et al., 2021).

Beta rhythm activity also plays an integral role in shooting performance. Similar to alpha, beta power is more pronounced in the left hemisphere, particularly in the anterior temporal area of professionals (Janelle et al., 2000; Loze et al., 2001). Comparisons between competitive and non-competitive states reveal a significant linear correlation between shooting accuracy and beta power in anterior frontal, central, temporal, and occipital regions (Zhang et al., 2021). Additionally, research on pre-performance rest states indicates that functional connectivity in the left hemisphere between sensorimotor and temporal cortices within the 13–20 Hz (beta-1) range is significantly correlated with subsequent shooting performance, suggesting that higher resting-state connectivity predicts better results (Gong et al., 2017).

Theta wave activity, in turn, is closely linked to focused concentration and performance success. Experienced shooters exhibit higher theta power (6–7 Hz) than novices across all periods, with the greatest difference observed during the aiming phase, reflecting intense concentration in experts (Haufler et al., 2000). In professionals, theta power at the Fz electrode shows a significant increasing trend in the 6 s before a successful shot, but follows an “inverted U-shaped” pattern (initial rise followed by a decrease) before failed attempts (Wang et al., 2023). An increase in theta power consistently occurs in the final second before the shot across performance groups (Cheng et al., 2023). However, a negative correlation between theta power in frontal and mid-occipital regions has been reported in novices, suggesting less efficient coordination (Zhang et al., 2021). Comparisons of competitive and non-competitive states also reveal a significant correlation between shooting accuracy and theta power in several brain regions (Zhang et al., 2021). Moreover, the characteristic path length of brain networks in the theta range during rest shows a significant linear correlation with subsequent shooting performance, further emphasizing the relevance of theta-related functional organization (Gong et al., 2017). Sensorimotor rhythms (SMR) serve as another important indicator of optimal motor preparation. Research on skilled air-pistol shooters indicates higher SMR power at the central area (Cz) in the final second before a shot, reflecting reduced extraneous sensorimotor cortex activity, which is associated with superior performance. This phenomenon is accompanied by reduced high-alpha coherence between frontal and left temporal regions (Fz-T3), signifying suppressed verbal-analytical interference at the crucial moment (Cheng et al., 2017). Differences also emerge among professionals when grouped by performance level: a successful group demonstrates higher SMR power at the central area (Cz) 3 s before the shot compared to a worse-performing group (Cheng et al., 2023).

Finally, delta rhythm is primarily discussed in the context of novice performance. An increase in averaged delta power has been observed before missed shots in novices, suggesting a distinct neural state associated with less efficient motor preparation (Badarin et al., 2023).

## 4 Cardiac activity

The primary tool for assessing cardiac function in sport shooting performance is ECG, also gathered through complementary devices, such as heart rate monitors (Ware et al., 2021). From ECG recordings, derives several key indicators of cardiac function, including heart rate, the duration of RR intervals, and the timing of shot delivery within the cardiac cycle. These measures form the basis for evaluating HRV, a widely used metric reflecting autonomic regulation. Common HRV indices include the standard deviation of NN intervals (SDNN), which captures overall variability, and the root mean square of successive differences (RMSSD), which reflects short-term, high-frequency variations and is considered an indirect marker of parasympathetic activity (Berntson et al., 2005). The RR interval itself, defined as the time elapsed between two successive R waves, is primarily used to estimate instantaneous heart rate (Lanfranchi and Somers, 2011).

The relationship between heart rate and shooting performance appears to be modulated by the athlete's level of expertise. In novices and individuals without systematic training experience, heart rate does not change significantly nor substantially affect shooting outcomes (Badarin et al., 2023). However, a different pattern emerges among elite shooters. One study found that successful shots are preceded by a decrease in heart rate and a corresponding increase in RR interval length, whereas no such changes occur during unsuccessful attempts (Wang et al., 2023). This discrepancy likely reflects the more refined autonomic regulation developed through extensive practice. Interestingly, heart rate in professional shooters does not differ between training and competition conditions, suggesting a degree of autonomic stability regardless of contextual demands (Loh et al., 2024).

Beyond heart rate dynamics, researchers have also examined the precise timing of shot execution within the cardiac cycle. Early work by Konttinen et al. (2003) compared shooting conditions (dry firing) with non-shooting conditions (aiming only) and found that trigger pulls are distributed evenly across the entire RR interval during aiming-only trials. In contrast, when actual shooting occurs, shots are delivered more frequently between 10% and 50% of the RR interval and less frequently between 50% and 90%. Notably, shots fired at the beginning and end of the interval (0%–50% and 70%–90%) demonstrate above-average accuracy, while those fired in the middle (50%–70%) yield poorer results. A subsequent study by Mets et al. (2007) confirmed that shooters tend to fire more often within the 10%–15% portion of the RR interval, but found no direct relationship between shot timing and accuracy. More recently, Gallicchio et al. (2019) reported a correlation between success rates and cardiac phase in professional shooters, with the highest accuracy observed between 20% and 30% of the RR interval. The authors proposed that this effect may be mediated by ballistic recoil: the phase of the cardiac cycle influences the body's stability at the moment of firing, making certain phases more favorable than others. Thus, shot accuracy may depend, at least in part, on whether the trigger is pulled during phases incompatible with the peak of the pulse pressure wave.

HRV has emerged as a particularly informative marker of autonomic regulation in shooters. Studies comparing skill levels indicate that elite shooters exhibit higher RMSSD values than their non-elite counterparts, suggesting greater parasympathetic nervous system activity (Zhao et al., 2024). SDNN, which reflects overall autonomic activity, also tends to be higher in more skilled shooters, although differences do not always reach statistical significance (Thompson et al., 2015). Collectively, these findings point to HRV as a potential predictor of shooting performance. For instance, HRV is significantly positively correlated with self-efficacy and actual performance, and more experienced shooters demonstrate lower heart rates in the preparatory period before shooting (Ortega and Wang, 2018). Furthermore, the magnitude of HRV changes—reflecting the dynamic balance between sympathetic and parasympathetic activation—is associated with shooting accuracy. Specifically, smaller fluctuations in HRV correlate with better marksmanship, implying that shooters whose autonomic nervous system is less reactive to stress are capable of more accurate performance (Thompson et al., 2015).

The interplay between cardiac activity and postural stability has also been investigated. Konttinen et al. (1998) demonstrated that heart rate decreases before firing in both elite and non-elite shooters with prior shooting experience. However, the pattern of this deceleration differs between groups. In non-elite shooters, a more pronounced heart rate reduction occurs during periods of stable rifle holding, which the authors attribute to intensive visual fixation on the target. In contrast, elite shooters exhibit heart rate suppression specifically when rifle stability is compromised, interpreted as a mobilization of psychomotor regulation to correct posture. This distinction highlights how autonomic responses become increasingly integrated with task-specific motor demands as expertise develops.

## 5 Oculomotor activity

Vision and visual skills constitute a fundamental component of precise shooting performance. Research consistently demonstrates that shooters exhibit superior visual capabilities compared to non-shooters, with further distinctions emerging among athletes of different skill levels. For instance, elite shooters demonstrate better accommodation scores than their non-elite counterparts, highlighting the role of visual efficiency in expert performance (Mon-López et al., 2022).

The study of oculomotor activity in shooting relies on several established measurement techniques. EOG enables the recording of the cornea-positive standing potential relative to the back of the eye, providing a means to assess eye movements through saccade analysis, which reveals gaze direction and movement amplitude. This method requires the placement of electrodes on the skin near the eyes (Creel, 2019). An alternative approach is eye tracking, which monitors gaze direction and focal point using a specialized camera without the need for electrode placement (Punde et al., 2017). Both methods yield valuable ocular parameters, including blink rate and a specific metric known as QE, which are thought to reflect the shooter's attentional focus and serve as potential biomarkers of shooting success.

Blink rate represents one of the more straightforward oculomotor indicators. Professional shooters tend to blink less frequently than less skilled individuals, with gaze stability correlating positively with increased concentration and reduced cognitive load (Kruchinina and Polikanova, 2025). Interestingly, changes in blink rate during shooting are not observed in novices, likely reflecting the absence of a systematic behavioral routine that professionals develop through training (Badarin et al., 2023).

The concept of QE has emerged as a particularly informative measure in shooting research. QE is defined as the final fixation of gaze on the target before shot execution, lasting at least 100 milliseconds within a 3° viewing angle (Kruchinina and Polikanova, 2025). Its onset occurs prior to the decisive stage of the action, while its offset is marked by gaze deviation from the target exceeding 3° for a duration of 100 milliseconds (Vickers and Williams, 2007). QE is widely regarded as a key indicator of shooting efficiency, reflecting an individual's capacity to sustain visual attention and mental concentration under pressure (Kruchinina and Polikanova, 2025). Consequently, QE duration and timing have been identified as predictors of shot success (Sögüt et al., 2025). The measurement of QE is typically accomplished using EOG, eye tracking, or video recording techniques.

The nature of the competitive environment significantly influences gaze behavior in professional shooters. In a study by Vickers and Williams (2007), two conditions were simulated: low-pressure shooting, where participants were informed that the study focused on gaze fixation, and high-pressure shooting, which involved the presence of a coach, selection consequences, and prize incentives. Additionally, participants performed bicycle ergometry at varying intensities—55%, 70%, 85%, and 100% of maximal oxygen consumption—to examine the interaction between physiological load and visual focus. The results revealed that longer QE duration was generally associated with higher accuracy. At lower exertion levels, shorter QE appeared sufficient to support accuracy, suggesting automated skill execution. However, at maximal intensity, maintaining a longer QE with sustained external focus on the target became essential for preventing performance deterioration, a phenomenon (Baumeister, 1984) defined as “choking”—performance decrement under circumstances that increase the importance of successful execution. Athletes who increased QE duration under pressure sustained high accuracy, whereas those who did not experienced sharp performance declines. This finding underscores the protective function of prolonged target fixation against the negative effects of anxiety, fatigue, and pain under extreme conditions.

Skill-related differences in QE parameters have been consistently documented. Gonzalez et al. (2017) demonstrated that experienced archers exhibit both earlier QE onset and longer QE duration compared to novices. Similarly, Vardar and Senduran (2021) compared elite and novice shooters across dry-fire and live-fire conditions, with dry shots serving as focus-practice trials without performance evaluation. Novices displayed significantly shorter QE during dry shots compared to successful live shots, whereas elite shooters showed no significant difference between conditions. This finding indicates that elite athletes maintain consistent focus regardless of situational demands, reflecting a more robust attentional system. The earlier QE onset observed in elite shooters suggests that they have developed more efficient strategies for extracting critical information from the visual scene, thereby enabling the transmission of higher-quality commands to the motor system. It is worth noting that the optimal QE duration for elite performers varies depending on the specific motor task, indicating that QE is not a fixed parameter but rather adapts to task constraints (Vickers, 2016).

## 6 Muscle activity

The assessment of muscular function in sport shooting by continuous EMG monitoring provides critical insights into muscle activation patterns during task execution. EMG serves as the primary method for recording bioelectrical activity in muscles, capturing the electrical currents generated during contraction through electrodes placed on the skin surface (De Luca, 2006). Complementary biochemical methods, such as lactate concentration measurement from blood samples, are employed to assess muscle fatigue, although this approach has limitations: it cannot provide real-time monitoring during performance and reflects systemic rather than muscle-specific changes (Cifrek et al., 2009). Together, EMG and lactate assessment offer a comprehensive view of muscle state and function, revealing not only overt performance characteristics but also subtle errors invisible to visual observation, such as excessive muscle tension that impedes smooth trigger manipulation despite a seemingly correct stance (Svecova et al., 2017).

In air rifle shooting, particular attention is directed toward the musculature of the upper limbs, including the trapezius, deltoid, triceps brachii, brachioradialis, and palmaris longus muscles (Kim, 2016; Konttinen et al., 1998). Konttinen et al. (1998) conducted EMG analysis of the trapezius, deltoid, brachioradialis, and palmaris radialis muscles in rifle shooters and observed distinct patterns between skill levels. Non-elite shooters exhibited a progressive increase in EMG activity during the 6 s preceding trigger pull, whereas elite shooters maintained stable, low-level activation. Higher EMG activity was directly associated with poorer rifle stability in both groups, though this relationship was considerably stronger among non-elite athletes. Notably, EMG levels alone could not predict shot success but were interpreted as reflecting psychomotor regulatory processes.

The distribution of muscular load between the upper extremities is inherently asymmetrical due to the mechanical demands of rifle support. The left arm assumes primary responsibility for bearing the rifle's weight, necessitating sustained tonic activity in the biceps brachii, forearm musculature, and trapezius. However, empirical evidence indicates that when tension exceeds an optimal threshold, rifle stability deteriorates, leading to increased weapon displacement. Comparative analysis of skilled and novice shooters reveals distinct neuromuscular recruitment patterns. Svecova and Vala (2016) found that experienced shooters exhibit minimal activation in the left biceps brachii while maintaining complete relaxation in the right forearm, with phasic muscle activity occurring exclusively during reloading. In contrast, inexperienced shooters demonstrate elevated left biceps tension accompanied by lateral rifle drift, coupled with inappropriate activation of the right biceps. This excessive grip force generation directly interferes with smooth trigger manipulation, thereby degrading shot execution.

The trapezius and deltoid muscle groups display load-dependent activation patterns that evolve over the course of a shooting session. Kim (2016) proposed dividing the shooting experiment into three temporal periods and found that shooting performance reached its highest scores during the middle period, a result attributed to muscular adaptation to the sustained load.

Muscle fatigue represents another important factor influencing shooting quality and is assessed using both EMG and lactate concentration measurements. Fatigue is understood as a long-term process commencing with exercise onset and progressing to the point of task failure (De Luca, 1984; Cifrek et al., 2009). EMG offers the advantage of continuous monitoring of local muscle fatigue during performance, as the biochemical and physiological changes accompanying fatiguing contractions are reflected in myoelectric signal properties (De Luca, 1984). Common EMG parameters for fatigue assessment include root mean square, an amplitude measure reflecting the level of muscle activation, and mean power frequency and median frequency, spectral parameters that capture frequency characteristics related to muscle fiber physiology and fatigue development (Bilodeau et al., 2003).

The susceptibility of specific muscle groups to fatigue varies according to shooting discipline. Della Santa et al. (2020) analyzed the impact of low-energy vs. high-energy cartridges on biceps brachii activity during shotgun shooting and observed progressive declines in median frequency and mean power frequency throughout the shooting sequence, substantiating the development of neuromuscular fatigue. This fatigue effect was more pronounced when participants used high-energy cartridges. In air pistol shooting, fatigue has been documented in the deltoids, biceps, brachioradialis, and trapezius muscles by the end of a shooting session (Kim, 2016).

Despite these observations, research on professional biathletes indicates that physiological fatigue does not necessarily compromise shooting accuracy. Heinrich et al. (2020) found that while fatigue increased overall shooting time, accuracy remained unaffected, suggesting that elite athletes develop compensatory mechanisms that prioritize precision over speed when necessary.

## 7 Postural balance

The ability to maintain steady posture during aiming is a fundamental characteristic contributing to shooting performance, as it provides the mechanical foundation for precise weapon alignment. The assessment of postural control in shooting contexts typically employs two complementary technologies: aiming trajectory tracking systems, such as the SCATT (Shooter's Coordination Analysis Target Training), which utilizes sensors installed on the weapon to monitor movement patterns, and force platforms, which measure body sway and weight distribution during stance (Mononen et al., 2007).

The relationship between postural balance and shooting accuracy is mediated through rifle stability. Insufficient balance control, particularly in the mediolateral plane, leads to increased oscillation of the rifle barrel, while anteroposterior body sway contributes to irregularities in the shooting trajectory (Ihalainen et al., 2016b). Thus, postural instability translates directly into weapon displacement, compromising the precision of shot placement.

Elite shooters demonstrate superior stability compared to their less skilled counterparts (Konttinen et al., 1998). However, recent evidence suggests that the role of body stabilization remains significant even among highly trained athletes. Research on elite performers confirms that postural balance continues to correlate with shooting performance not only in between-subject comparisons but also across individual shots within a single athlete's series (Lang and Zhou, 2022). This finding indicates that even at the highest levels of expertise, variations in postural control contribute to performance fluctuations. The persistence of this relationship in elite athletes may be attributed to the highly refined nature of their skills: less successful shots are more likely to arise from subtle variations in balance rather than from gross technical errors.

The impact of postural stabilization is considerably more pronounced in novice shooters. Era et al. (1996) demonstrated that shot quality is directly dependent on stability, with body oscillations being significantly greater during poorer shots compared to more accurate ones. This observation underscores the fundamental role of postural control in the early stages of skill development.

Ihalainen et al. (2016a) conducted a comprehensive analysis of six components of shooting technique: aiming time, stability of hold, total shot time, cleanliness of triggering, aiming accuracy, and triggering timing. Among these, four factors—stability of hold, cleanliness of triggering, aiming accuracy, and triggering timing—were identified as the most important determinants of shooting performance. Each of these parameters was found to improve with increasing shooter qualification, establishing a clear link between expertise and technical proficiency. Notably, the results elucidated the indirect nature of postural balance contributions: while the direct effect of balance on accuracy is minimal, its influence on rifle stability is substantial. Postural control thus serves as a foundational element that enables the effective execution of more direct performance determinants.

## 8 Respiratory activity

The regulation of breathing represents a critical physiological component affecting precision shooting performance. To record respiratory activity during shooting, researchers typically employ special belt-shaped sensors that detect chest expansion and compression (Badarin et al., 2023). Additional assessment methods include measurement of vital capacity and respiratory muscle strength, which provide complementary information about pulmonary function (Ertürk et al., 2023; Karaduman et al., 2022). Together, respiratory rate, vital capacity, and respiratory muscle strength serve as biomarkers for evaluating performance success in shooting contexts.

Patterns of respiratory activity vary across different types of shooting sports and skill levels. In archery, experienced athletes begin to exhale during the aiming and shooting phases, and a significant correlation between breathing and shooting performance emerges only in this experienced group. In contrast, inexperienced archers exhibit higher breathing rates during the shooting phase, suggesting less efficient respiratory regulation (Mohamed et al., 2014).

In the context of air rifle shooting, distinct respiratory patterns have been documented. Badarin et al. (2023) observed that overall respiratory rate does not change significantly in novices throughout the experimental session. However, instantaneous respiratory rate is higher before a hit than before a miss, and a decrease in respiratory rate is typically observed immediately before the shooting phase. These findings indicate that even at novice levels, spontaneous respiratory adjustments may influence shot outcomes.

The relationship between respiratory capacity and shooting performance has been systematically investigated. Karaduman et al. (2022) demonstrated a positive correlation between shooting performance, respiratory muscle strength, and pulmonary function, with shooters achieving the highest performance scores also exhibiting the greatest respiratory capacity. Extending these findings, Ertürk et al. (2023) discovered a significant positive correlation between shooting scores and vital capacity measures in air rifle athletes. Notably, these relationships varied by both gender and competition level. Respiratory capacity emerged as a meaningful predictor of performance for elite, junior, and female shooters, with vital capacity explaining a substantial portion of the variance in their shooting scores. In contrast, no significant relationship between shooting performance and vital capacity was observed for male participants, suggesting that other factors, such as dynamic balance, may play a more determinant role in this subgroup.

Beyond static capacity measures, the temporal coordination of breathing with shot execution appears important. In air rifle shooting, breathing control facilitates synchronization of the shot with the exhalation phase, a period when heart rate naturally decreases. Ortega and Wang (2018) showed that experienced shooters consciously employ breathing as part of their psychophysiological regulation before the shot, a strategy that correlates with higher HRV and better performance outcomes. This integration of respiratory control into pre-shot routines underscores the role of breathing as an active regulatory mechanism rather than a passive physiological process.

## 9 Biological feedback and future perspectives

Precision shooting performance depends on the coordinated activity of multiple physiological systems. Cortical, cardiac, oculomotor, muscular, postural, and respiratory processes each contribute to successful performance, and their integration distinguishes experts from novice performers. Together, these findings underscore that shooting performance is not determined by any single physiological measure but rather by the coordinated interaction of multiple systems. The most significant works, divided into sections by biomarkers, are listed in Table 1.

**Table 1:** Biomarkers of performance in shooting sports and their highlighted characteristics.

Biomarker	Participants	Equipment	Results	References
Brain activity				
Power of delta, theta, and alpha bands	21 novices	Air rifle	Delta and alpha activity power (right temporal lobe) were significantly lower before a hit than before a miss. Theta activity (frontal and central regions) significantly negative correlates with shooting performance.	Badarin et al., 2023
Power of alpha, beta bands, and SMR	18 shooting students; 3 elite shooters	Air pistol	The success of the shot correlates with an increase in alpha activity in the left hemisphere (T7) and specific dynamics of the theta rhythm (Fz) 6 s before the shot. In case of failure these patterns are absent. The SMR range and the right temporal region (T8) remain inactive.	Wang et al., 2023
Power of alpha, beta bands	18 elite shooters, 10 novices	Air pistol	Elite shooters exhibited lower ERP amplitudes across the entire scalp (alpha/beta bands) than non-elites, while high performance was linked to higher ERS amplitudes in left central, right posterior parietal	Del Percio et al., 2009
Cardiac activity				
Heart rate, length	18 shooting students; 3 elite shooters	Air pistol	The heart rate and R-R interval for a successful shot showed a significant decrease and increase, respectively, 6 s before the shot; no significant changes occurred for an unsuccessful shot.	Wang et al., 2023
Heart rate	12 shooters: elite shooters, non-elite shooters	Small-bore	A decrease in heart rate before shooting was observed in all shooters. No connection was found between shooting performance and heart rate dynamics.	Konttinen et al., 1998
RRI, placement of shot in RRI, heart rate	13 experienced biathletes	Small-bore	The best shots were fired less frequently between 200 and 300 ms, more frequently between 500 and 600 ms after the R-wave, under exercise conditions: less frequently between 100 and 200 ms after the R-wave and between 20% and 30% of the R-R interval.	Gallicchio et al., 2019
HRV parameters	20 elite and 25 non-elite	Air pistol	More experienced shooters had lower anxiety scores and were more self-confident; their RMSSD, TP, and HFn were higher. LFn was lower.	Zhao et al., 2024
Oculomotor activity				
QE	10 elite archers; 11 non-elite archers, 10 novices	Bow	An earlier onset of the QE and a longer duration were observed in expert shooters than in novices. A longer QE is associated with better results.	Gonzalez et al., 2017
QE	8 shooters: experience >10 years and experience < 2 years	Air pistol	Higher accuracy was observed when firing real shots. Elite shooters demonstrated a longer eye rest period when firing dry fire. No differences were found when firing real shots.	Vardar and Senduran, 2021
Muscle activity				
Upper body muscle activity	12 shooters: elite shooters, non-elite shooters	Small-bore	Non-elite shooters: muscle activity increased prior to the shot and correlated with instability. Elite shooters: muscle activity was lower, more stable, and less strongly associated with performance.	Konttinen et al., 1998
Upper body muscle activity	4 sport shooters, experience: 6.25 ± 1.71 years	Air pistol	Aiming activates the trapezius, deltoid, and triceps; preparation activates the biceps and brachioradialis. At the end of shooting, fatigue occurs in the deltoids, biceps, brachioradialis, and trapezius.	Kim, 2016
MNF, MDF	3 licensed shooters	Shotgun	MNF and MDF decreased during firing. Fatigue was more pronounced when using high-energy cartridges.	Della Santa et al., 2020
Postural balance				
Rifle stability	12 shooters: elite shooters, non-elite shooters	Small-bore	Elite shooters demonstrated superior rifle stability. There was a correlation between performance and stability: the better the stability, the higher the score.	Konttinen et al., 1998
Postural stability, Rifle stability	4 sport shooters, experience: 6.25 ± 1.71 years	Air pistol	The vertical displacement of the pistol was greater than the horizontal. The most significant changes in the body's center of mass were observed in all directions at the initial stage. The greatest postural stability was achieved in the middle stage.	Kim, 2016
Indicators of successful shot	40 athletes: 19 international level, and 21 national level	Air rifle	Good postural balance was strongly correlated with a more stable rifle grip. International-level athletes demonstrated significantly better results compared to national-level athletes.	Ihalainen et al., 2016a
Physical and coordination parameters	11 elite, 9 juniors	Air rifle	A negative correlation was found between shooting performance and dynamic balance of the right leg in men. A positive correlation was found between shooting performance and expected vital capacity in women.	Ertürk et al., 2023
Respiratory activity				
Respiratory rate	21 novices	Air rifle	The overall frequency did not change during the experiment; the instantaneous breathing rate was higher before a hit, and a decrease in breathing rate was observed before the shooting phase.	Badarin et al., 2023
MIP, MEP, FVC, FEV_1_, FEV_1_/FVC, SVC, MVV	167 police cadets	Pistol	A positive correlation was found between shooting performance and MIP and MEP and between shooting results and FVC, FEV1, SVC, and MVV.	Karaduman et al., 2022
Vital capacity of the lungs	11 elite, 9 juniors	Air rifle	Elite shooters: a positive correlation was found between shooting performance and expected vital capacity. Juniors: between shooting results and actual and percentage vital capacity of the lungs and hand grip strength.	Ertürk et al., 2023

FEV_1_, forced expiratory volume in 1 second; FVC, forced vital capacity; HFn, high-frequency (normalized units); HRV, heart rate variability; LFn, low-frequency (normalized units); MDF, median frequency; MEP, minimal expiratory pressure; MIP, maximal inspiratory pressure; MNF, mean frequency; MVV, maximal voluntary ventilation; QE, quiet eye; RMSSD, root mean square of successive differences; RRI, RR interval; SMR, sensorimotor rhythms; SVC, slow vital capacity; TP, total power.

Given that these biomarkers can be measured and assessed, substantial research has been directed toward developing training interventions aiming to accelerate skill acquisition and enhance performance. The influence of training on brain activity is evident in longitudinal observations of novice shooters, who demonstrate increased alpha power in the left temporal lobe as performance improves, indicating the emergence of automated processing and reduced reliance on verbal-analytical strategies (Kerick, 2004). Vision training programs have been shown to improve shooting accuracy, reaction time, and gun kinematics in skeet shooters, underscoring the plasticity of visual skills (Guo et al., 2024). Postural stability can be enhanced through targeted interventions, including vibration platform training combined with unstable surfaces, which significantly reduces vertical barrel oscillation (Hung et al., 2021). Respiratory patterns are amenable to modification through controlled breathing techniques, with low-frequency breathing training showing tendencies toward improved shooting accuracy in young athletes (Kovaleva et al., 2014). Attentional control can be enhanced through brief meditation interventions, which improve target focus, reduce distraction, and increase dorsolateral prefrontal cortex activity (Gao and Zhang, 2023).

Among training approaches, those utilizing biological feedback have garnered particular attention lately. Neurofeedback, which provides real-time information about brain activity, enables athletes to learn voluntary regulation of cortical oscillations (Corrado et al., 2024). SMR-based neurofeedback has demonstrated superior efficacy compared to alpha-based training in inexperienced pistol shooters (Gong et al., 2020). However, contradictory evidence suggests that alpha oscillation-based training targeting the left temporal lobe positively correlates with shooting performance, indicating that efficacy may depend on protocol specifics (Lo et al., 2025). Integrated approaches combining temporal and left parietal regions have also shown performance improvements (Kakhaki and Taheri, 2017). Frontal theta-based neurofeedback, while not producing statistically significant performance differences, has been subjectively reported to improve concentration (Toolis et al., 2023).

Recent methodological innovations include composite indices such as the Comprehensive Oscillatory State Modulation Index (COSMI), which integrates SMR, theta, and high-beta rhythms. Increases in this index reflect more efficient cortical processing and have been linked to improved cognitive and motor performance in shooters (Chen et al., 2025). Infra-low frequency neurofeedback, targeting slow cortical potentials below 0.1 Hz, has demonstrated significant positive effects on shooting performance by facilitating a state of calm alertness (Bakhtafrooz et al., 2025). Beyond neurofeedback, HRV and respiratory biofeedback aim to optimize autonomic regulation, enabling athletes to remain calm and precise under pressure (Kakhaki and Taheri, 2017). An innovative direction involves delivering biofeedback *in situ*, integrated directly into the shooting task. Behneman et al. (2012) employed EEG and ECG signals to control a vibrating device that permitted shot execution only when ideal readiness levels were achieved, demonstrating accelerated skill acquisition in archers.

The growing complexity of data obtained from these multimodal systems has driven the adoption of advanced analytical tools, particularly machine learning, to uncover meaningful patterns and enhance feedback precision. The application of machine learning addresses the challenge of processing and analyzing large volumes of physiological signals acquired during training.

Applications of machine learning in shooting research have progressed from general signal processing toward specific performance-relevant questions. Sögüt et al. (2025) employed hybrid CNN-LSTM architectures to automatically detect QE periods from EOG signals, achieving superior accuracy compared to traditional threshold-based methods and enabling real-time feedback on gaze stability. Guru et al. (2025) applied explainable AI techniques to HRV features, demonstrating that shot outcomes in archery could be predicted with moderate accuracy, with SHAP (SHapley Additive exPlanations) analysis identifying RMSSD and SDNN as the most influential predictors across individual athletes. Flyckt et al. (2023) utilized Vision Transformer architectures on skeleton tracking data from multiple body sensors, achieving up to 80% accuracy in classifying shooting phases and identifying that forearm angle, head position, and body alignment were the most critical kinematic features distinguishing successful from unsuccessful shots. Almeida et al. (2023) developed a hybrid neural-fuzzy model (MANFIS) that enables novice shooters to rapidly adjust their stance toward experienced shooter profiles in simulator environments. These examples illustrate a progression from prediction toward interpretability—a crucial step for translating algorithmic outputs into actionable coaching recommendations. However, the number of studies combining machine learning with multimodal physiological data in shooting remains limited, highlighting an important gap for future investigation.

## 10 Discussion

The psychophysiological literature on precision shooting has established a set of measurable biomarkers across neural, cardiac, oculomotor, muscular, postural, and respiratory systems. Collectively, these findings indicate that elite performance emerges from the coordinated interaction of multiple physiological systems rather than from any single optimal state. It is important to note, this interaction appears to be synergistic rather than additive: for example, the simultaneous optimization of respiratory phase and HRV produces a stabilizing effect on posture that exceeds the sum of their individual contributions. At the same time, examination of the literature reveals several contradictions and open questions that deserve attention. Across all physiological domains reviewed substantial individual variability is evident. This variability is not merely measurement noise but reflects different strategies for achieving successful performance, supporting the need for personalized training approaches.

Some apparent contradictions in the literature become clear when viewed through the lens of individual adaptation. The hypothesis of neural efficiency posits a general reduction of cortical activation with expertise (Li and Smith, 2021) contradicts findings of local increases in alpha synchronization and SMR power before successful shots (Wang et al., 2023). However, this contradiction disappears when we understand that efficiency involves a selective redistribution rather than a uniform decrease: experts suppress task-irrelevant networks (e.g., verbal-analytic left temporal regions) while enhancing task-relevant networks (e.g., sensorimotor and attentional networks). Similarly, conflicting data on the optimal heart rate phase for shooting—from 10%–50% to 20%–30% of the RR interval (Konttinen et al., 2003; Mets et al., 2007; Gallicchio et al., 2019)—likely reflect differences in physical exertion, shooting discipline, and individual baseline autonomic tone. The results of neurofeedback training studies also appear inconsistent: Gong et al. (2020) demonstrated that SMR-based neurofeedback significantly outperformed alpha-based training in pistol shooters, whereas Lo et al. (2025) reported that left-temporal alpha enhancement positively correlated with shooting performance, contradicting the earlier finding of SMR superiority. Toolis et al. (2023) found that frontal theta-based neurofeedback produced no statistically significant performance improvements, though athletes subjectively reported enhanced concentration. Meanwhile, integrated protocols combining multiple frequency bands have yielded mixed outcomes across different populations, with some studies reporting positive effects (Kakhaki and Taheri, 2017) and others failing to replicate these findings. These discrepancies, however, can be explained by a single principle: the most effective training method depends on the athlete's baseline EEG profile, not on a universal protocol. Therefore, the observed inconsistencies are not a lack of evidence, but rather represent empirical evidence for the fundamentally individual nature of psychophysiological optimization. This conclusion supports the development of personalized training approaches.

This conclusion has direct implications for the prioritization of physiological biomarkers as a function of athletic proficiency. Our synthesis reveals that the predictive contribution of each biomarker is not fixed but shifts systematically with expertise, supporting a hierarchical model of biomarker relevance. In this framework, peripheral, readily observable systems dominate early learning, whereas central neuro-autonomic systems provide the marginal gains that distinguish expert from superior performance (Figure 3).

**Figure 3:**
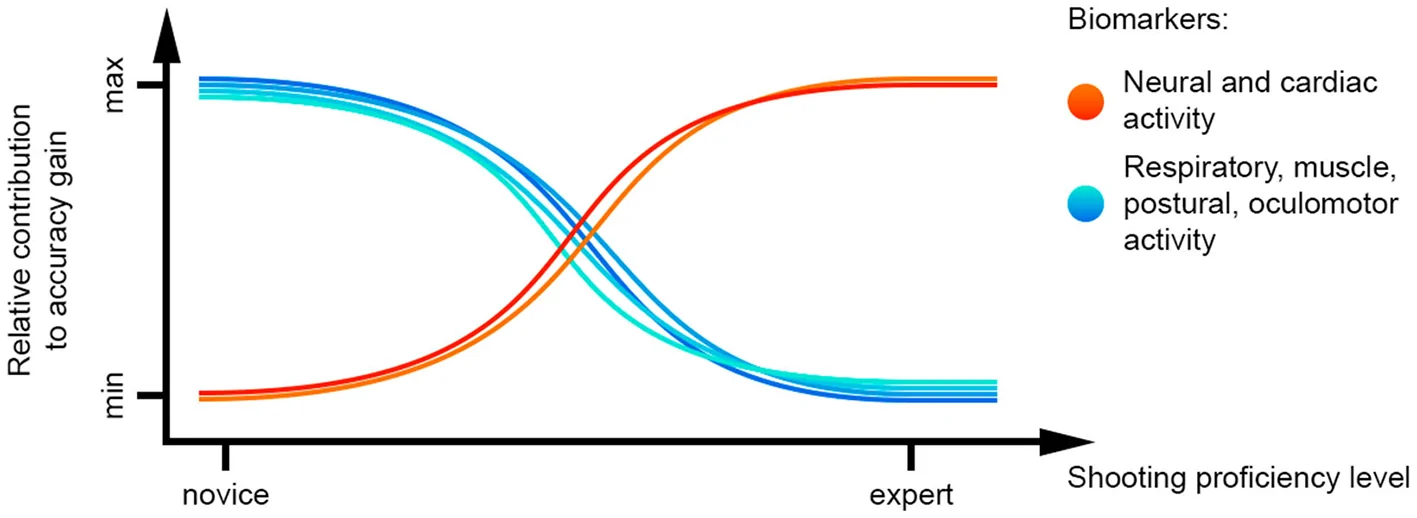
Schematic representation of the physiological biomarkers contribution to shooting performance prediction as a function of expertise. Peripheral systems (respiration, posture, oculomotor, and muscle) provide the largest performance gains during early skill acquisition but plateau at elite levels. Central systems (neutal, cardiac) contribute minimally in novices, but become the primary discriminators of performance in experts, reflecting the shift from gross motor regulation to fine autonomic and cortical optimization.

In novice shooters, performance variability is primarily driven by gross motor and regulatory deficits. Respiratory patterns are erratic and poorly synchronized with shot execution (Badarin et al., 2023); postural sway is pronounced and directly translates into score fluctuations (Era et al., 1996; Mononen et al., 2007); QE durations are often insufficient for stable target acquisition (Vardar and Senduran, 2021); and muscle activity exhibits a progressive, maladaptive increase in tension rather than the stable low-level activation characteristic of experts (Konttinen et al., 1998). Critically, cardiac and cortical measures show weak or inconsistent relationships with trial-by-trial performance in this population (Badarin et al., 2023; Zhang et al., 2021). Thus, the most accessible performance gains in novices arise from normalizing respiratory, postural, oculomotor, and muscular patterns—systems that offer a wide “window of improvement” and respond robustly to basic technical training. Introducing neurofeedback or HRV biofeedback at this stage would likely be premature, as the signal-to-noise ratio is unfavorable and athletes lack the physiological stability required to benefit from fine-grained autonomic self-regulation.

Conversely, in elite shooters, peripheral systems have been compressed to near-ceiling levels through years of deliberate practice. Respiratory-postural coupling, QE duration, and muscle activation profiles show minimal fluctuation between successful and unsuccessful shots (Gonzalez et al., 2017; Konttinen et al., 1998). Under these conditions, residual performance variance is no longer explained by gross technical factors but by subtle, centrally mediated regulatory processes. Here, cardiac and cortical biomarkers emerge as primary discriminators. HRV indices, particularly RMSSD and SDNN, reflect parasympathetic tone and predict performance outcomes in expert cohorts (Zhao et al., 2024; Thompson et al., 2015). The timing of trigger pull within the cardiac cycle distinguishes optimal from suboptimal shots (Gallicchio et al., 2019). Cortical dynamics—including left-temporal alpha synchronization (suppression of verbal-analytic interference), frontal-midline theta (sustained concentration), and SMR power (motor preparation)—show consistent, trial-specific relationships with performance exclusively in skilled athletes (Wang et al., 2023; Cheng et al., 2017; Chen et al., 2025). In this population, central biomarkers provide the marginal gains that separate champions from finalists, and neurofeedback interventions yield measurable improvements precisely because the athlete's peripheral “scaffolding” is already established (Gong et al., 2020; Lo et al., 2025).

This expertise-dependent shift has significant consequences for both experimental design and applied practice. If confirmed through longitudinal multimodal studies, it would imply that the optimal sequence of biomarker inclusion in predictive models depends critically on the participant's proficiency level. For novices, a parsimonious model incorporating respiratory rate, postural sway, QE duration, and EMG activity would capture the majority of performance variance, with cardiac and EEG parameters adding little predictive value. For experts, the inverse holds: models must prioritize high-resolution cardiac and EEG features, while peripheral measures serve primarily as covariates to control for baseline state. This sequential, expertise-moderated prediction aligns with broader calls for multimodal approaches that respect the hierarchical organization of psychophysiological regulation (Badarin et al., 2023; Chen et al., 2025).

Importantly, this hierarchy is not static but reflects the athlete's position on the learning curve. As novices progress toward expertise, the performance bottleneck shifts upward: respiratory and postural control reach asymptote first, followed by oculomotor and muscular efficiency, with cardiac and cortical variability becoming the primary leverage points only at later stages. This trajectory suggests that biofeedback protocols should be periodized—initially targeting broad, observable systems using simple feedback (e.g., breathing pace, balance indicators), and progressively introducing more abstract neuro-autonomic feedback as the athlete's physiological baseline stabilizes. Such an approach would maximize training efficiency and reduce the risk of overwhelming athletes with complex, low-yield signals during early skill acquisition.

Building on this hierarchical perspective, several observations about the current state of the literature inform future research directions. Most studies have employed cross-sectional expert-novice comparisons, which provide valuable descriptions of expertise-related differences—such as higher HRV, longer QE durations, and distinct EEG profiles in elite shooters (Zhao et al., 2024; Gonzalez et al., 2017)—but do not capture how these differences emerge over time. Longitudinal training studies are needed to address this gap. The majority of investigations have examined individual physiological systems in isolation. However, shooting is inherently integrative: respiratory exhalation slows heart rate, which influences postural sway and rifle stability, which in turn affects gaze control. Multimodal studies, such as that by Badarin et al. (2023), have shown that missed shots are preceded by convergent changes across multiple domains, supporting the value of integrated measurement approaches. Ecological validity remains a consideration. The QE has been extensively studied under controlled conditions, and its protective function becomes particularly critical under high-pressure, high-fatigue states that resemble competition (Vickers and Williams, 2007). This supports the importance of simulating realistic task demands.

The above considerations point to several important directions for future research. First, the predominance of single-system studies limits understanding of how all biological processes interact during shooting performance. Multimodal approaches that capture the integrated nature of physiological regulation are needed to reveal cross-system interactions and provide a more complete picture of the physiological state underlying successful performance. Longitudinal training studies combining repeated multimodal assessments with targeted interventions would help establish causal relationships between specific biomarker changes and performance improvements. Second, substantial inter-individual variability observed across biomarker domains necessitates the development of personalized feedback protocols, tailored to individual athletes' baseline physiological profiles and specific performance deficits. Third, composite indices that integrate multiple biomarkers, such as the COSMI (Chen et al., 2025), offer a pathway toward holistic performance monitoring, although such indices require validation across different shooting disciplines and skill levels. Fourth, the integration of machine learning and explainable artificial intelligence into training platforms holds particular promise: future systems may not only predict performance outcomes but also provide real-time, interpretable feedback to athletes and coaches, highlighting which specific physiological or postural parameters require adjustment, thereby bridging the gap between complex data analytics and practical, actionable guidance. Finally, the development of portable sensor suites for use in authentic training environments, combined with real-time monitoring and feedback systems, will be essential for translating laboratory-based findings into practical applications in sport settings.

Several limitations of the present review should be acknowledged. First, the review does not employ a systematic or quantitative meta-analytic approach, which limits the ability to assess the magnitude and consistency of effects across studies. Second, sample sizes in many of the reviewed studies are relatively small due to the highly specialized nature of the topic, which limits statistical power and generalizability. Nevertheless, all included studies reported statistically significant results (*p* < 0.05) according to their respective analytical frameworks. Third, the literature reviewed is predominantly cross-sectional, comparing experts and novices at a single time point. Such designs cannot distinguish between pre-existing traits that predispose individuals to excel in shooting and neurophysiological changes that emerge as a direct consequence of training. Fourth, heterogeneity of experimental paradigms, equipment, and outcome measures across studies complicates direct comparisons and synthesis of findings. Fifth, the majority of studies have been conducted under controlled laboratory conditions that may not fully capture the psychophysiological demands of actual competition, including the effects of environmental stressors, audience presence, and performance pressure. Despite these methodological constraints, we considered it important to provide a comprehensive overview of the current state of research in this emerging field.

## 11 Conclusion

This review synthesizes psychophysiological biomarkers of shooting performance across neural, cardiac, oculomotor, muscular, postural, and respiratory systems. Our findings highlight that elite performance emerges from the coordinated interaction of multiple systems. The observed expertise-related patterns—such as left-hemisphere alpha synchronization, increased HRV, prolonged QE duration, and stable low-level muscle activation—offer quantifiable targets for biofeedback interventions. A critical gap remains: most studies are cross-sectional and single-modal. Future multimodal, longitudinal research combining explainable artificial intelligence with real-time physiological monitoring could transform training personalization. Such advances have the potential to accelerate skill acquisition, reduce injury risk from maladaptive tension patterns, and optimize performance under competitive pressure. Implementing these evidence-based biomarkers into portable feedback systems would bridge laboratory findings and practical sports medicine applications.
